# Biogeographic Distribution Patterns and Their Correlates in the Diverse Frog Fauna of the Atlantic Forest Hotspot

**DOI:** 10.1371/journal.pone.0104130

**Published:** 2014-08-20

**Authors:** Tiago S. Vasconcelos, Vitor H. M. Prado, Fernando R. da Silva, Célio F. B. Haddad

**Affiliations:** 1 Departamento de Ciências Biológicas, Universidade Estatual Paulista, Bauru, São Paulo, Brazil; 2 Departamento de Zoologia, Universidade Estadual Paulista, Rio Claro, São Paulo, Brazil; 3 Departamento de Ciências Ambientais, Universidade Federal de São Carlos, Sorocaba, São Paulo, Brazil; University of Sao Paulo, Brazil

## Abstract

Anurans are a highly diverse group in the Atlantic Forest hotspot (AF), yet distribution patterns and species richness gradients are not randomly distributed throughout the biome. Thus, we explore how anuran species are distributed in this complex and biodiverse hotspot, and hypothesize that this group can be distinguished by different cohesive regions. We used range maps of 497 species to obtain a presence/absence data grid, resolved to 50×50 km grain size, which was submitted to *k*-means clustering with *v*-fold cross-validation to determine the biogeographic regions. We also explored the extent to which current environmental variables, topography, and floristic structure of the AF are expected to identify the cluster patterns recognized by the *k*-means clustering. The biogeographic patterns found for amphibians are broadly congruent with ecoregions identified in the AF, but their edges, and sometimes the whole extent of some clusters, present much less resolved pattern compared to previous classification. We also identified that climate, topography, and vegetation structure of the AF explained a high percentage of variance of the cluster patterns identified, but the magnitude of the regression coefficients shifted regarding their importance in explaining the variance for each cluster. Specifically, we propose that the anuran fauna of the AF can be split into four biogeographic regions: a) less diverse and widely-ranged species that predominantly occur in the inland semideciduous forests; b) northern small-ranged species that presumably evolved within the Pleistocene forest refugia; c) highly diverse and small-ranged species from the southeastern Brazilian mountain chain and its adjacent semideciduous forest; and d) southern species from the Araucaria forest. Finally, the high congruence among the cluster patterns and previous eco-regions identified for the AF suggests that preserving the underlying habitat structure helps to preserve the historical and ecological signals that underlie the geographic distribution of AF anurans.

## Introduction

Dividing the world or large geographical regions into meaningful biological units has long been of general interest for macroecologists and biogeographers. For instance, the evaluation of the world's zoogeographical regions proposed by A. R. Wallace more than 100 years ago is still a subject of recent studies (e.g., [1]). While early biogeographical regions were generated based on researchers' knowledge of species distribution (e.g., the original zoogeographical regions proposed by Wallace), recent regionalization proposals have been performed by considering a large amount of species information available on digital databases coupled with the use of one or several quantitative statistical methods (e.g., [1], [2]). Irrespective of what method is used, a species assemblage within a determined biogeographic region can be expected to share a large amount of history with other assemblages within the region, but relatively little with those in other biogeographic regions [3]. For this reason, biogeographic regions may be viewed as operational species pools [3], which provide fundamental abstractions of the geographical organization of life in response to past or current physical and biological forces. Regionalization schemes thus provide spatially explicit frameworks for answering many basic and applied questions in historical and ecological biogeography, evolutionary biology, systematics, and conservation [2], [4].

Biogeographic regionalizations in South America have mainly been performed at a global scale perspective, and have relied on a variety of methods and biological models (see examples and references in [1], [2]). These schemes either consider the whole continent as a distinct biogeographic unit (e.g., Neotropical region *sensu* Wallace's zoogeographical classification) or split the continent into two or three regions depending on either the methodological approaches or the biological traits among taxa (e.g., dispersal capability) [1], [2], [5]. All else being equal, the scale of analysis is an important factor in determining the final number of regions. For instance, global analyses using similar clustering methods always identify Europe as part of the Palaeartic region [1], [2], but scaling down the analysis to the continent level generates a more refined identification of sub-regions [6]. In South America, a cluster analysis was performed in order to devise a regionalization system based on amphibian distribution. In the analysis, the authors recognized four biogeographic regions for the group [7]. Specifically, although some areas of the seasonally dry Atlantic Forest were grouped within the savanna-like vegetation cluster, the authors found that most of the area encompassed by the Atlantic Forest hostspot (*sensu* [8]) is considered to be a biogeographic unit for the South American amphibians [7]. Here, we devise a regionalization scheme for the current original extent of the Atlantic Forest hotspot (i.e., without considering habitat loss by recent deforestation that occurred during the last century) in order to explore how amphibians are distributed throughout this complex and biodiverse domain, and then to generate a map of amphibian diversity focused on the composition of regional faunas within the hotspot.

Amphibian species of the Atlantic Forest (hereafter AF) are a highly diverse group, and their morphological structures, behavioral repertoires, and breeding strategies are greatly diversified as well. For instance, there are approximately 550 anuran species from the AF that exhibit 39 different reproductive modes, most of which are endemic at the species, genus, or even family level [9], [10]. This high diversity of reproductive modes is attributed to the successful utilization of the diversified and humid microhabitats present in this biome [9]. Yet, the gradient of species richness and number of reproductive modes is not randomly distributed, so there is a parallel of increased species richness and number of reproductive modes between dry/seasonal and evergreen humid forests [11], [12]. There is also a great number of micro-endemic species associated with the Atlantic coast, some of which have been reported at only one location [9]. Because of this high anuran diversity associated with different patterns of species richness and concentrations of micro-endemic species, we hypothesize that the anuran distribution within the AF can be distinguished by different cohesive regions, thus consisted of different species pools.

Specifically, our first goal is to determine the number and the spatial position of these regions using a cluster analysis. Then, based on preview studies that showed that richness gradients and range size of species are differently distributed throughout the hotspot [9], [11]–[13], we hypothesize that gradients of climatic conditions, topographic variations, and habitat structures are non-mutually exclusive conditions that determine cohesive regions within the AF. Thus, because the patterns of species distributions are ultimately determined by the rates of speciation, extinction, and dispersal [14], [15], and because physiological constraints and limited dispersal are two key characteristics of most amphibian species, we hypothesize three potential explanations for the cohesive anuran regions in the AF. The first hypothesis considers the well-known fact that larger ranges in elevation promote speciation through habitat specialization and altitudinal isolation, which increases endemism and, consequently, the discrepancy in species richness between sites within a region [9], [16]–[18]. Therefore, we first hypothesize that topography could be one of the determinants of the anuran biogeographic regions, because regions with extensive variation in topography would harbor small-ranged species due to historically limited dispersal capabilities and would thus increase the chance of higher speciation rates at these areas. The second hypothesis considers the fact that energy- and humidity-related variables have been shown to be key environmental determinants of the richness and composition of amphibian communities [11], [12], [19]. Due to the wide latitudinal variation in the AF, our second hypothesis is that climate may be a strong predictor of the anuran biogeographic regions identified by our cluster analysis. Finally, Rueda and collaborators [6] showed that the habitat structure in Europe has a strong influence on the identification of biogeographic regions for different taxa (including anurans). Thus, considering the fact that the habitat provides the templet on which evolution forges animal life-history strategies (the concept of habitat templet [20], [21]), our third hypothesis is that the anuran's cohesive regions can be recognized as a consequence of the vegetation distribution within the AF. We also used deviance partitioning techniques to disentangle the relative influence of each predictor and to identify the independent and shared influences of topography, climate, and vegetation structure on the identified anuran biogeographic sub-regions within the AF hotspot.

## Materials and Methods

### Study area

**Figure 1:**
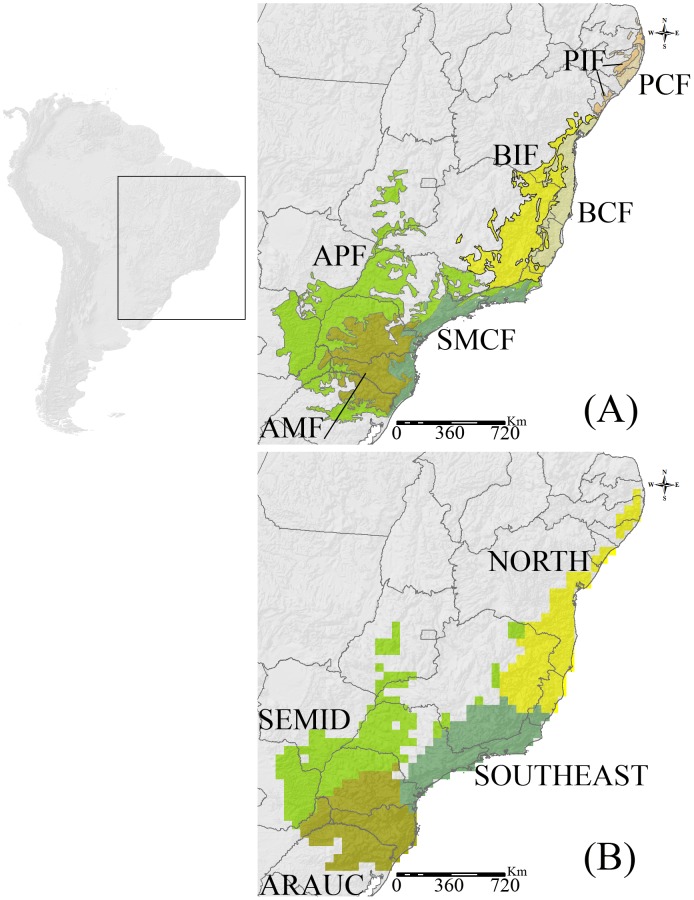
Major Atlantic Forest eco-regions, modified from the World Wildlife Fund designations (A): AMF = Araucaria Moist Forests; APF = Alto Paraná (semideciduous/deciduous) Forests; BCF = Bahia Coastal (moist) Forests; BIF = Bahia Interior (semidecidual/decidual) Forests; PCF = Pernambuco Coastal (moist) Forests; PIF = Pernambuco Interior (semideciduous) Forests; and SMCF = Serra do Mar Coastal (moist) Forests. Biogeographic regions based on the anuran fauna generated through *k*-means clustering with *v*-fold cross-validation (B).

Characterized by a complex topography (elevation varies from sea level to 2,000 m a.s.l.) and a wide latitudinal distribution along the Brazilian Atlantic coast (latitudinal distribution of *c.* 25°), the AF hotspot is considered one of the world's most species-rich, yet notoriously endangered and understudied ecosystems [8], [22]. There are many classifications attributed to the AF (e.g., [23]), and one of the most commonly used [24] divides the domain in terms of its floristic composition, landscape, and climatic attributes into the categories of open, dense, and mixed ombrophilous/evergreen forest, which are widely distributed throughout the Brazilian coast, but the mixed forest (also known as the Araucaria forest) is mainly found along the southern rim of the hotspot [23], [24]; the seasonally dry forest is also known as semideciduous and deciduous forests, and it is characterized by the partial and total loss of leaves, respectively, as a result of the pronounced precipitation seasonality over the year (Figure 1A). Although they also have wide latitudinal distributions, deciduous and semideciduous forests are located in inland areas that are mostly located in northeastern and southeastern Brazil [23], [24].

To devise a new amphibian regionalization scheme, we considered the current original extent of the AF (i.e., deforestation has not been considered herein, see [24]) provided by the Conservation International portal (http://www.conservation.org/where/priority_areas/hotspots/Documents/CI_Biodiversity-Hotspots_2011_ArcView-Shapefile-and-Metadata.zip). We then divided the AF into 469 grids at *c.* 50×50 km grain size, considering that each grid was covered by at least 50% of the AF. Finally, we were able to construct a presence/absence matrix based on the anuran distribution, which was then submitted to cluster analysis.

### Species distribution data

There are currently 543 amphibian species in the AF hotspot, 529 of which are anurans [10]. Here, we excluded the grids in which small natural patches of the AF do not cover at least 50% of the biome. Consequently, we were unable to consider the species restricted to these small patches, such as the narrowly-ranged species *Adelophryne baturitensis*, *A. maranguapensis*, *Bokermannohyla diamantina*, and *B. itapoty*. Island-endemic species, such as *Scinax alcatraz* and *S. faivovichi*, have also been excluded from the analysis. In the end, a total of 496 species (∼94% of AF anurans) were considered for the regionalization process (Table S1).

Almost all species range maps were obtained from the International Union for Conservation of Nature (IUCN) portal (http://www.iucnredlist.org/technical-documents/spatial-data), and the amphibian nomenclature was updated according to the Amphibian Species of the World 5.6 portal [25]. The species that were not available in the database (e.g., recently described species, such as *Brachycephalus pulex* and *B. toby*) had their maps created in ArcGIS 10.1 considering their original descriptions. The rasterized range maps were overlaid onto each grid cell to generate a presence/absence matrix. Although they possess some level of error [26], range maps represent the areas where a particular species can be expected to occur, and it will be expected to be found only in suitable habitats within these areas [6]. Thus, overprediction is an inherent methodological limitation of these kinds of range maps [27]. Within a macroecological perspective, however, they may function very well at grains greater than 50×50 km [28]. That is to say, although the IUCN amphibian maps might include either over- or underpredictions [26], using range maps is presumed to be as reliable as more refined information regarding a given species distribution (e.g., point occurrence records from survey data and/or herpetological collections) if the goal is to document broad-scale patterns of species distribution [28].

### Environmental and topographic variables

Five abiotic variables (one topographic and four climatic variables) were gathered and averaged for each grid cell. Annual precipitation (P), precipitation seasonality (PS), and mean annual temperature (T) were obtained from the WorldClim database at a 10×10 km resolution [29]. Annual actual evapotranspiration (AET), a measure of water-energy balance, was also obtained at a 10×10 km resolution at http://www.fao.org/geonetwork/srv/en/metadata.show?id=37233. The standard deviation of elevation, a measure of topographic heterogeneity (TOP), was calculated for each grid cell based on elevation data (∼1×1 km resolution) available at https://lta.cr.usgs.gov/GTOPO30. All of these variables are known to represent either potential physiological limits for amphibians or as barriers to dispersal, and they are closely associated with species richness patterns of both plants and animals [11]–[12], [19], [30].

### Regionalization procedure

First, *k*-means clustering combined with *v*-fold cross-validation was applied to the presence/absence matrix [31], [32]. The classical *k*-means clustering algorithm requires the number of clusters (*k*) to be established in advance, and utilizes a subset of *k* random initialization cells that are treated as the initial cluster centers, and then proceeds as a two-step iterative procedure in which cluster centres and clusters are successively recalculated. The first step starts with the assignment of each cell to its nearest cluster center in terms of species compositional distance, herein considered as the Hellinger distances [33]. In the second step, each cluster center is updated by making it equal to the mean of the cells assigned to it. The process is repeated (we used 50 iterations) so that the cluster and cluster centers change in each replicate, and they converge to a locally optimal position in the data space [6], [7]. The *k*-means clustering technique was combined with *v*-fold cross-validation in order to obtain the optimal number of clusters based on species composition without regard to the spatial proximity of the grids (see [6]). In summary, the algorithm determines the “best” number of clusters within a range of pre-determined cluster numbers (we set these from two to 25 clusters). The *k*-means clustering technique with *v*-fold cross-validation was performed using Statistica (StatSoft).

### Correlates of cluster patterns

Considering the fact that the regionalization procedure we used is designed to generate biotic regions based on differences in species assemblages affected by complex interacting factors [5], [6], we explore the extent to which topography, climate, and the vegetation structure of the AF are expected to identify the cluster patterns recognized by *k*-means clustering. Because the climatic and topographic variables were gathered at higher resolutions than those of the ∼50×50 km AF grid, we averaged all values of these variables within each AF grid cell, thus balancing out the different data scales inherent in each independent variable. The vegetation structure was considered based on the major AF eco-regions from the World Wildlife Fund designations [34] (Figure 1A) and was used as a multinomial variable to evaluate the extent to which animal species composition is associated with the AF habitat structure (e.g., [6]).

Because the dataset is linearly distributed (visually checked by means of partial residual plots graphic, [35]; Figure S1), we followed Rueda and collaborators' study [6] and performed Generalized Linear Models (GLMs) with multinomial logit-link for modelling a multinomial response variable (i.e., the present *k*-means cluster solution) as a function of one or more continuous predictors. Due to different magnitudes of measurement of each predictor and in order to facilitate the interpretation of the regression coefficients, all predictor variables were standardized to have a mean of zero and a standard deviation of 1.0 prior to analysis [36]. Collinearity among the predictors was verified by the Variance Inflation Factor (VIF; [37]) and we considered them to be not strongly collinear (VIF<5.1).

We generated several single- and multiple-variable explanatory models that could potentially explain the cluster patterns: a) full multiple-variable model that considered all predictors; b) a climatic and eco-regional multiple-variable model that considered the AF eco-regions and climatic variables; c) a climatic and topographic multiple-variable model that considered climate and the standard deviation of elevation, which is presumed to generate high levels of endemism in the AF (see [9]); d) a topographic and eco-regional multiple-variable model that considered the AF eco-regions and the standard deviation of elevation; e) a climatic multiple-variable model that considered only climatic variables; f) an eco-regional multiple-variable model that considered only the AF eco-regions; and g) a topographic single-variable that considered only the standard deviation of elevation. The model selection approach was based on the lowest Akaike Information Criterion (AIC; [38]). We considered the best model to be the one based on the lowest AIC required to partition the deviance of each response variable into independent effects of a particular predictor and co-varying effects of two or more predictors that cannot be disentangled [6], [39].

We also performed a Principal Component Analysis (PCA) of all 469 grid cells in order to visualize the patterns of distribution of the abiotic characteristics and the clusters that represented each grid cell [40].

## Results

The cluster analysis identified four biogeographic regions in the AF based on anuran species composition (Figure 1B). Cluster 1 (hereafter SEMID) is located in AF inland areas, and it encompasses most of the semideciduous forest and transitional areas to the Cerrado (i.e., the Brazilian savanna-like vegetation). Cluster 2 (hereafter SOUTHEAST) is comprised of the coastal AF in southeastern Brazil, where most of the area falls within the ombrophilous forest and adjacent areas of semideciduous forest. Cluster 3 (hereafter NORTH) encompasses the northeastern Brazilian semideciduous/deciduous and ombrophilous forests, and cluster 4 (hereafter ARAUC) is mostly congruent with the Araucaria forest in southern Brazil (Figure 1B).

Among all models of predictor variables, the full model (with all variables included) was the best one for explaining the cluster patterns (Table 1). This model explained higher levels of variance (80.10%) than the models comprised solely of climatic, topographic, or eco-regional variables (Table 1). However, the magnitude of the regression coefficients of the full model shifted in terms of their importance in explaining the variance for each cluster patterns (Table 2). The variable temperature and precipitation seasonality were the strongest predictors of the SEMID and ARAUC clusters, but this relationship was positively and negatively associated with these clusters, respectively (Table 2). That is, while warmer temperatures and precipitation seasonality predict the former cluster, cooler temperatures and more homogeneous rainfall predict the ARAUC cluster. Positive temperature is the only predictor of the NORTH cluster, whereas cooler temperatures, rough topography, AET, and precipitation seasonality are significant predictors of the SOUTHEAST cluster (Table 2).

**Table 1:** Generalized Linear Models of amphibians' *k*-means group in the Atlantic Forest.

Rank	Model	k	AIC	w_i_	Pseudo R_2_
1	Full Model	6	328.2	0.972	80.10
2	Climatic & Eco-regional Model	5	335.3	0.028	79.08
3	Climatic & Topographic Model	5	470.5	0	66.26
4	Climatic Model	1	576.4	0	57.56
5	Topographic & Eco-regional Model	2	615.5	0	55.93
6	Eco-regional Model	1	689.1	0	49.74
7	Topographic Model	1	1090.1	0	16.27

The models are sorted according to the lowest Akaike Information Criterion (AIC). k = number of the predictor variables included in the model; Pseudo R_2_ = coefficient of determination; w_i_ = evidence of 0.972 for the Model 1. See Methods for predictors' abbreviations.

**Table 2:** Regression coefficients of determination of the full multiple-variable Generalized Linear Model of amphibians' *k*-means group in the Atlantic Forest (eco-regional variables are omitted due to the lack of statistical significance with any cluster).

Clusters	TOP	AET	T	P	PS	ECOR
pFM	**0.004**	**0.023**	**0.000**	0.511	**0.000**	**0.000**
SEMID	−0.599	0.004	**2.120**	0.453	**1.360**	-
SOUTHEAST	**0.637**	**1.235**	**−0.950**	−0.197	**3.511**	-
NORTH	0.397	−0.349	**1.973**	−0.203	−0.423	-
ARAUC	−0.435	−0.890	**−3.143**	−0.053	**−4.449**	-

TOP = topography; AET = annual actual evapotranspiration; T = temperature; P = precipitation; PS = precipitation seasonality; ECOR = AF eco-regions; pFM = ANOVA p values of the full model. See Results for clusters' abbreviations. Significant coefficients (p≤0.01) are highlighted in bold.

**Figure 2:**
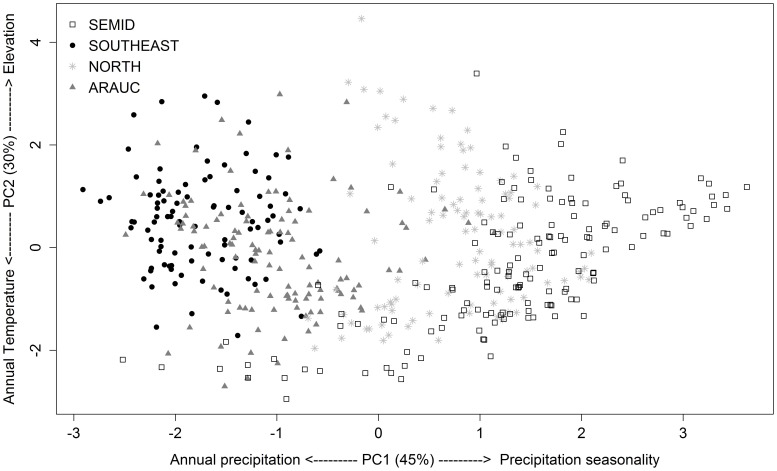
Principal component analysis (PCA) based on abiotic (annual precipitation, precipitation seasonality, mean annual temperature, annual actual evapotranspiration, and standard deviation of elevaton) and biotic (species richness and range size) variables of all 469 grid cells in the Atlantic Forest. Different symbols represent biogeographic regions based on the anuran fauna generated through *k*-means clustering with *v*-fold cross-validation.

The PCA results are represented in Figure 2 and shows that the first axis segregates both the SEMID and NORTH clusters from the other ones, since the SEMID and NORTH clusters are characterized by having higher precipitation seasonality than SOUTHEAST and ARAUC, which, in turn, have lower values of annual precipitation. The clusters overlap greatly at the second axis, but SOUTHEAST is slightly more commonly associated with rough topography than the other clusters are (Figure 2).

**Figure 3:**
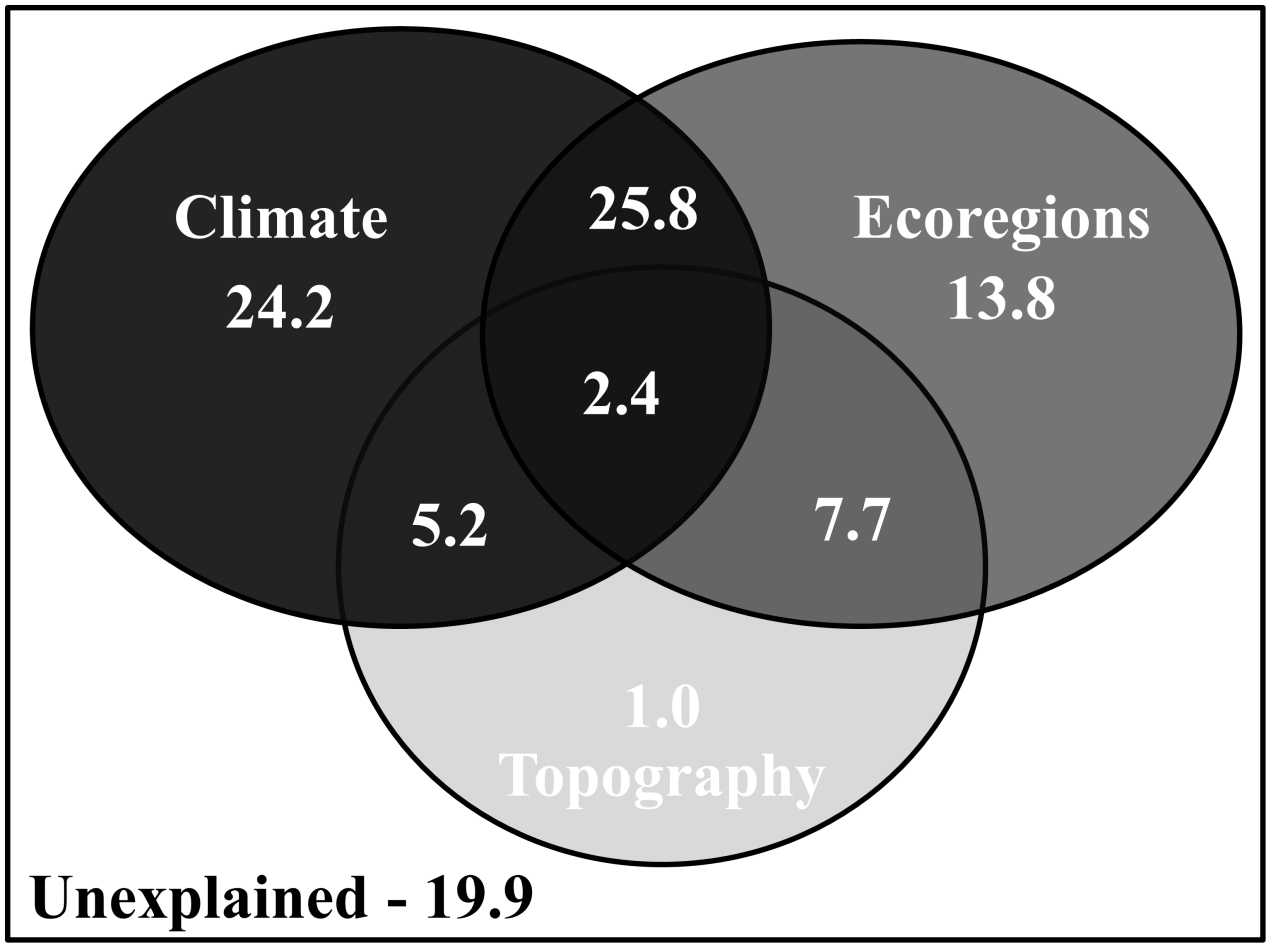
Deviance partitioning analysis representing the deviance in cluster configurations explained by climate (annual precipitation, precipitation seasonality, mean annual temperature, and annual actual evapotranspiration), vegetation structure of the AF (eco-regions considered in Figure 1A), and topography (range in elevation). The light-dark gradient of the figure represents the low-high deviance explained by the predictors.

The deviance partitioning indicates that a combined effect of the climate and vegetation structure of the AF (eco-regions) accounted for the largest fraction (25.8%) of the variability of the anuran cluster patterns identified herein (Figure 3). However, the largest independent effect is accounted for climate (24.2%), followed by the vegetation structure of the AF (13.8%) and topography (1.02%) (Figure 3).

## Discussion

The biogeographic patterns found for amphibians are broadly congruent with ecoregions identified in the AF, but their edges, and sometimes the whole extent of some clusters, present much less resolved patterns compared to the previous classification (e.g., [23], [24]). The SEMID and ARAUC clusters are broadly congruent with the southeastern Brazilian semideciduous and southern Araucaria forests, respectively (Figure 1A and 1B). On the other hand, the SOUTHEAST and NORTH clusters are consistent with a combination of subregions, mostly composed by ombrophilous and their adjacent semideciduous/deciduous forests.

The present study identified that climate, topography (i.e., the endemism-related variable), and the vegetation structure of the AF explained a high percentage of variance of the cluster patterns identified, a finding which agrees with previous studies that defined biogeographic regions for diverse taxa, including amphibians (e.g., [6], [7]). For instance, climate is well known to be strongly associated with broad-scale geographic patterns of species distributions [19], [30]. Therefore, it is reasonable that climatic gradients determined by the latitudinal variation in the AF are important forces in determining the present clusters (Table 2). Furthermore, as reported previously in Europe [6], the underlying vegetation structure of the AF is also considerably important for predicting the present cluster patterns, in which some of the clusters represent specific AF eco-regions (e.g., the Araucaria forest and the ARAUC cluster), while others represent a combination of eco-regions (e.g., SOUTHEAST). Indeed, it is well known that the water-energy balance is a strong correlate of plant distribution [6], [19], so it is not surprising that the shared effect of climate and AF vegetation distribution on the anuran biogeographic patterns was relatively high in the present study. Finally, although the topography accounts for only a small fraction of the variance of the identified cluster patterns, it is particularly important in predicting the SOUTHEAST cluster, which harbors the complex mountain chain in this region (see discussion ahead).

Considering a previous regionalization performed for South American amphibians [7], the identification of the SEMID cluster was already expected. The frog fauna from the inland semideciduous forest is made up of a mix of typical Cerrado and AF species, most of which are widely-ranged species [7], [41]. Hence, the most common feature shared by the SEMID species is the fact that they are both less diverse [11] and more widely distributed compared to species from other clusters (see also Figure S2 and Figure S3). This factor might be related to the fact that areas with minor topographic variation, such as the area encompassed by the SEMID cluster, favor population dispersal, and consequently, low speciation rates are expected in this area. This finding is reinforced by the fact that anurans from the semidecidual AF are more similar to the adjacent Cerrado anuran assemblages, which have more similar homogeneous topography and a harsher environment (higher temperatures and precipitation seasonality) than other AF ecoregions, such as the ombrophilous forest, which is more humid and which presents a rough topography [41]. Conversely, more homogeneous rainfall over the year and cooler temperatures are the strongest correlates of the ARAUC cluster. This is expected because, while variation in precipitation decreases, temperature markedly increases its seasonality at higher latitudes [42].

In NORTH, the only correlate identified was positive temperature. This is expected in a way, because the influence of positive temperatures becomes evident in the more northerly regions, closer to the Equator, where the climate is hotter. The SOUTHEAST cluster was correlated with almost all climatic variables analyzed, but precipitation seasonality was the strongest correlate. This finding was not expected, because this cluster mostly includes the ombrophilous forest, which is characterized by moist weather over the year, with no well-defined dry season [10], [23]. Thus, this unexpected correlation is more influenced by the presence of transitional areas of semidecidual forest in SOUTHEAST (see Figures 1A and 1B). The semidecidual forest considered in SOUTHEAST was likely not clustered with the SEMID because the anuran fauna of the semideciduous forest closer to coastal mountains includes some species that typically reside in the ombrophilous forest, and which is usually absent from those more distant and inland semideciduous forests (see examples in [41]). All other correlates in SOUTHEAST are expected: AET is known to be highly correlated with animal distribution (see [19]); and the negative correlation found between temperature and SOUTHEAST is probably due to cooler climate in this cluster than in NORTH and SEMID ones, particularly in areas where the mean temperature tends to decrease as the altitude increases (e.g., the southeastern Brazilian mountain chain).

Although the NORTH and SOUTHEAST clusters have different environmental predictors, these regions share interesting features in terms of anuran biogeographic patterns. In fact, they are recognized as “rich and rare” regions in South America for their amphibian diversity (i.e., they possess high species richness with restricted ranges; [13], see also Figure S2 and Figure S3). Due to different aims and methodological approaches, Villalobos and collaborators [13] considered the entire extent of the SOUTHEAST and NORTH clusters to be a continuous “rich and rare” region, but the identification of two distinct biogeographic species pools in the present study raises interesting questions regarding the evolution of amphibians in the AF. Although we found that climate, topography, and the vegetation structure of the AF are important in determining the present cluster patterns, we hypothesize that the recognition of two distinct micro-endemic species pools should result, at least partially, from the past climate history (e.g., the persistence of historically stable areas during the Pleistocene glaciations) and also from differences in topography along the extent of the AF (such as the presence of the complex mountain chains of Serra do Mar and Serra da Mantiqueira in the SOUTHEAST cluster). The extent of the NORTH and SOUTHEAST clusters agree with the predicted historical Pleistocene forest refugia (21,000 years before present; 21 ka BP), so these historically stable areas are expected to retain high levels of endemism for diverse taxa ([43] and references therein), including amphibians [13], [22], [44]. Moreover, the mountain chain in the southeastern Brazil is expected to favor the genetic diversification of amphibians, since it breaks the AF biome up into many small humid microhabitats and ultimately promotes speciation through geographic isolation [9]. This phenomenon has been found to be the case for amphibians [22], and high levels of endemism have also been reported for diverse taxa in SOUTHEAST ([43] and references therein). Therefore, although both NORTH and SOUTHEAST are recognized by their large numbers of small-ranged anuran species, we hypothesize that the different topography and the persistence of these areas over the course of the climate history (since the late Pleistocene) experienced by these clusters ultimately resulted in the evolution of two distinct species pools.

## Conclusions

In summary, we propose that the anuran fauna of the AF can be split into four biogeographic regions characterized by: a) less diverse and widely-ranged species that predominantly occur in the inland semideciduous forests, where the weather is hot and seasonally dry (SEMID); b) northern small-ranged species that presumably evolved/survived to extinction within the Pleistocene forest refugia, where the climate nowadays is hot (NORTH); c) highly diverse and small-ranged species from the southeastern ombrophilous and its adjacent semidecidous forest, where the climate is cooler (except when compared to ARAUC) and the topography is rough (SOUTHEAST); and d) southern species from the Araucaria forest, where the weather is cooler and the rains are well distributed throughout the year (ARAUC). The high congruence among the cluster patterns and previous eco-regions identified for the AF (Figure 1A and 1B) corroborates the habitat templet concept [20], [21], and suggests that preserving the underlying habitat structure (i.e., natural forest formations) helps to preserve the historical and ecological signals that underlie the geographic distribution of species [6], including the AF anurans. Nonetheless, it is important to emphasize that our regionalization scheme did not consider the human-induced deforestation that reduced the AF extension to ∼7% of its original distribution [10]. In addition, the herpetological literature is dynamic regarding the updating of the geographic ranges of the species and/or the description of new ones (e.g., [45], [46]). Thus, future regionalization schemes that consider the current remnants of the AF and the updated geographic ranges of the species after deforestation would be of interest for conservation biogeographers, who would be able to assess how much habitat loss can erase and/or maintain the broad-scale biogeographic patterns of AF anurans, mainly in areas with higher deforestation rates, such as the area encompassed by the NORTH cluster [22]. Finally, although other biological data (e.g., species traits and phylogenetic relationships) and the congruence of biogeographic patterns across multiple taxonomic groups are undoubtedly necessary for properly establishing conservation actions [7], [13], the regionalization process is an important step for identifying biogeographic regions that contain centers of origin, have been colonized by dispersing organisms, or have been subjected to large-scale forces such as the Pleistocene glaciations [47].

## Supporting Information

Figure S1 **Partial residual plots for each covariate in each cluster generated by the** ***k*** **-means clustering with** ***v*** **-fold cross-validation.**
(TIF)

Figure S2 **Geographical patterns of anuran amphibians range sizes (mean log_10_ range size of species at each grid cell) in the Atlantic Forest hotspot.**
(TIF)

Figure S3 **Anuran richness gradient in the Atlantic Forest hotspot.**
(TIF)

Table S1 **Anuran amphibians from the Atlantic Forest hotspot considered for the regionalization procedure (species are alphabetically sorted).**
(DOCX)
