# Supplementary figures and images for: Biogeographic Distribution Patterns and Their Correlates in the Diverse Frog Fauna of the Atlantic Forest Hotspot
Source: PLoS One. 2014 Aug 20;9(8):e104130. doi: 10.1371/journal.pone.0104130 (PMC4139199; doi:10.1371/journal.pone.0104130)

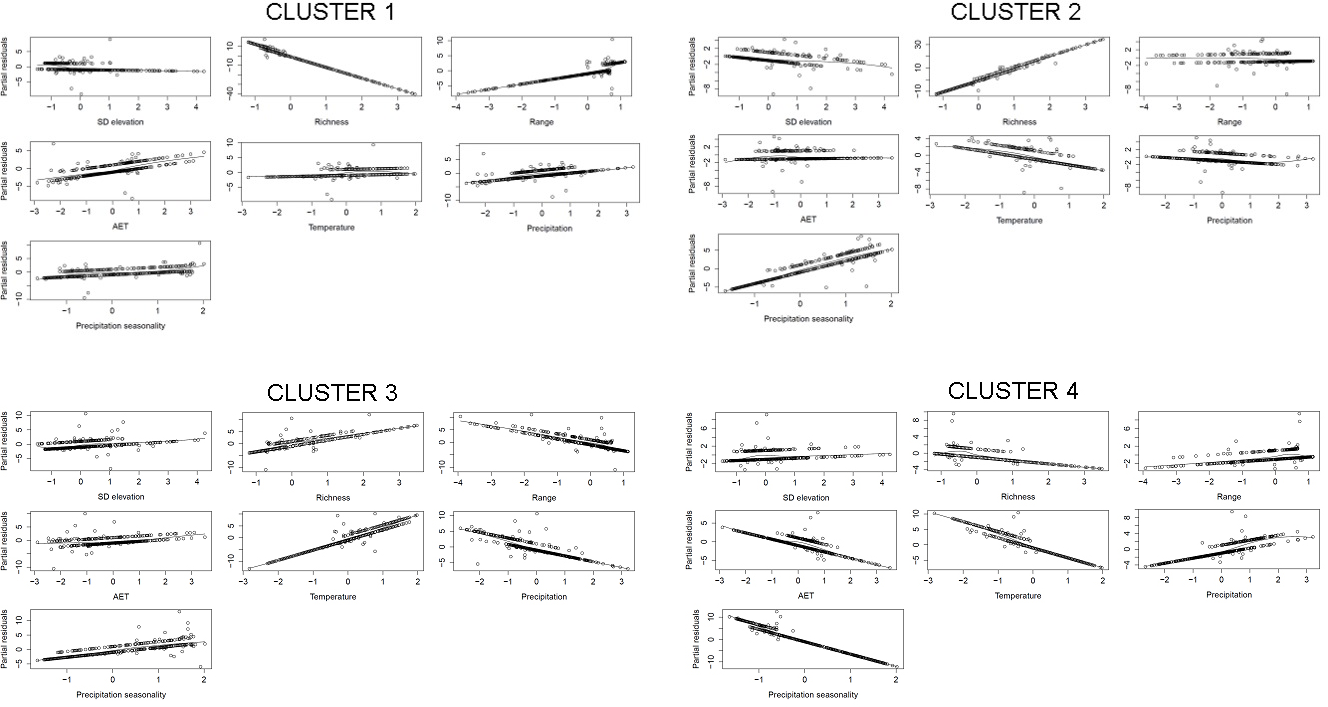

Supplement: Figure S1 — Partial residual plots for each covariate in each cluster generated by the k -means clustering with v -fold cross-validation. (TIF) [file pone.0104130.s001.tif]

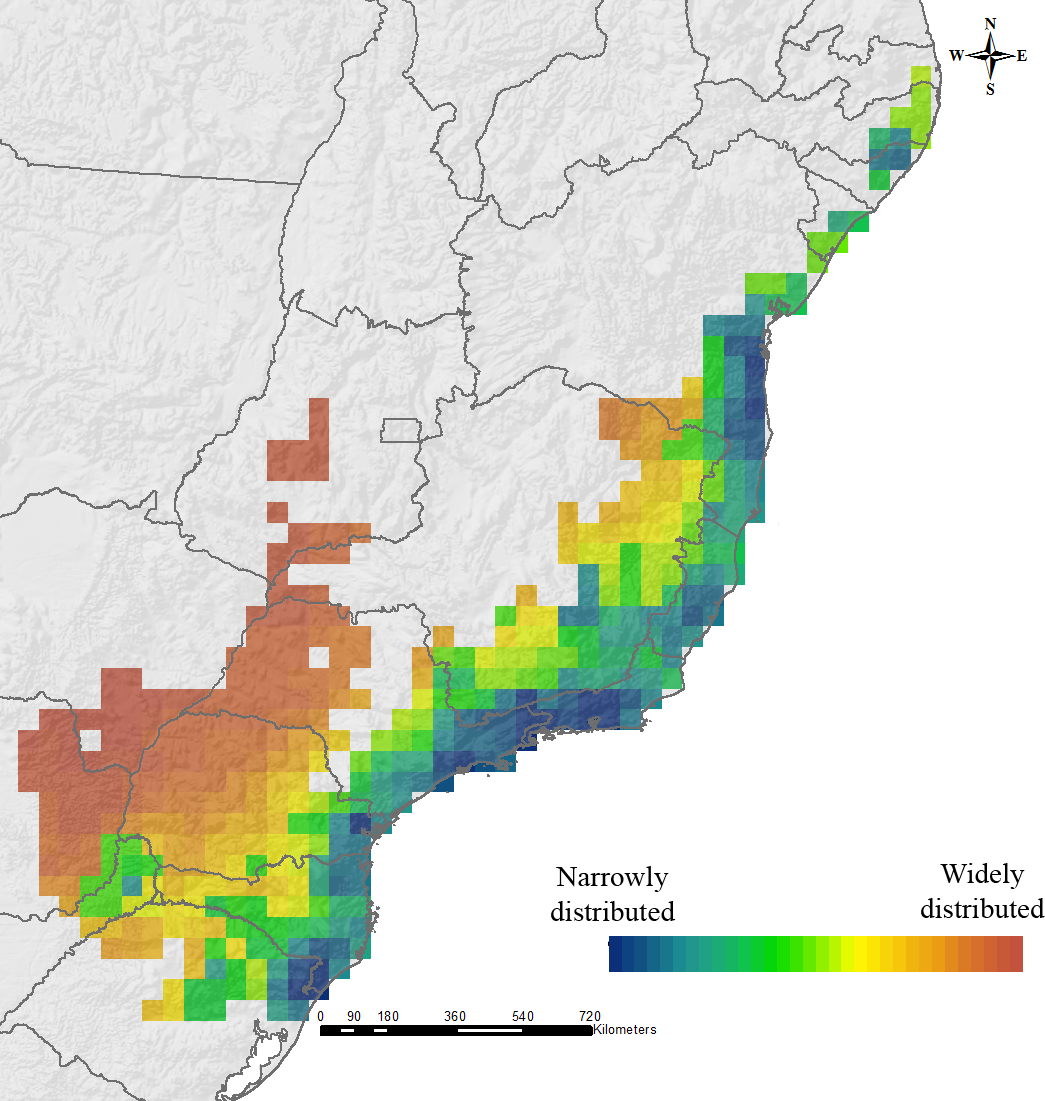

Supplement: Figure S2 — Geographical patterns of anuran amphibians range sizes (mean log10 range size of species at each grid cell) in the Atlantic Forest hotspot. (TIF) [file pone.0104130.s002.tif]

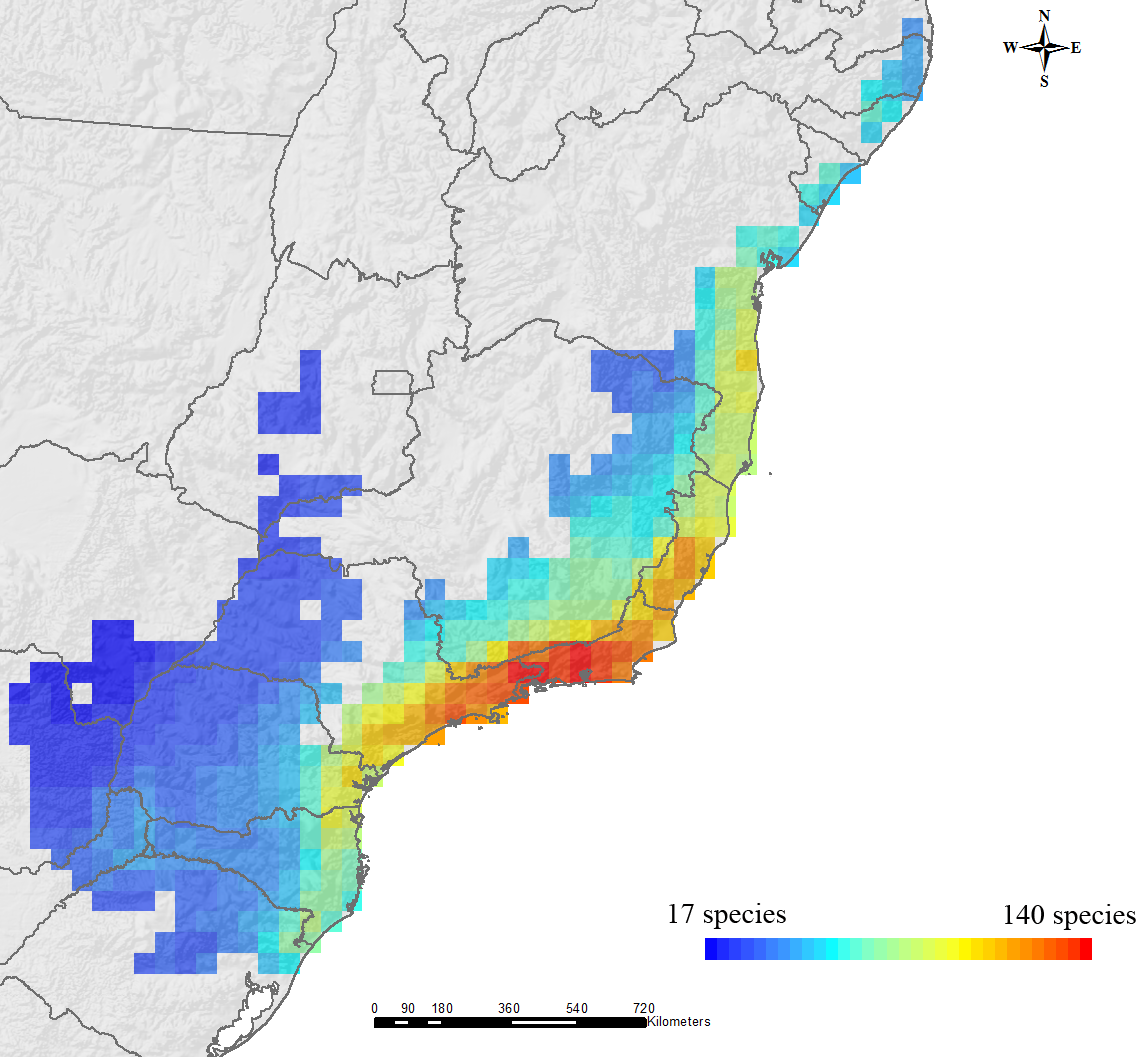

Supplement: Figure S3 — Anuran richness gradient in the Atlantic Forest hotspot. (TIF) [file pone.0104130.s003.tif]
