# Supplementary material for: Biogeographic Distribution Patterns and Their Correlates in the Diverse Frog Fauna of the Atlantic Forest Hotspot
Source: PLoS One. 2014 Aug 20;9(8):e104130. doi: 10.1371/journal.pone.0104130 (PMC4139199; doi:10.1371/journal.pone.0104130)
Supplement: Table S1 — Anuran amphibians from the Atlantic Forest hotspot considered for the regionalization procedure (species are alphabetically sorted). (DOCX) [file pone.0104130.s004.docx]

**Table S1**: Anuran amphibians from the Atlantic Forest hotspot considered for the regionalization procedure (species are alphabetically sorted).

| **Genus** | **species** |
| --- | --- |
| Adelophryne | mucronatus |
| Adelophryne | pachydactyla |
| Adenomera | ajurauna |
| Adenomera | araucaria |
| Adenomera | bokermanni |
| Adenomera | marmorata |
| Adenomera | nana |
| Adenomera | thomei |
| Agalychnis | aspera |
| Agalychnis | granulosa |
| Allobates | olfersioides |
| Aparasphenodon | arapapa |
| Aparasphenodon | bokermanni |
| Aparasphenodon | brunoi |
| Aplastodiscus | albofrenatus |
| Aplastodiscus | albosignatus |
| Aplastodiscus | arildae |
| Aplastodiscus | callipygius |
| Aplastodiscus | cavicola |
| Aplastodiscus | cochranae |
| Aplastodiscus | ehrhardti |
| Aplastodiscus | eugenioi |
| Aplastodiscus | flumineus |
| Aplastodiscus | ibirapitanga |
| Aplastodiscus | leucopygius |
| Aplastodiscus | musicus |
| Aplastodiscus | perviridis |
| Aplastodiscus | sibilatus |
| Aplastodiscus | weygoldti |
| Arcovomer | passarellii |
| Bokermannohyla | ahenea |
| Bokermannohyla | astartea |
| Bokermannohyla | capra |
| Bokermannohyla | caramaschii |
| Bokermannohyla | carvalhoi |
| Bokermannohyla | circumdata |
| Bokermannohyla | claresignata |
| Bokermannohyla | clepsydra |
| Bokermannohyla | feioi |
| Bokermannohyla | gouveai |
| Bokermannohyla | hylax |
| Bokermannohyla | ibitipoca |
| Bokermannohyla | langei |
| Bokermannohyla | lucianae |
| Bokermannohyla | luctuosa |
| Bokermannohyla | martinsi |
| Bokermannohyla | nanuzae |
| Brachycephalus | alipioi |
| Brachycephalus | atelopoide |
| Brachycephalus | brunneus |
| Brachycephalus | bufonoides |
| Brachycephalus | didactylus |
| Brachycephalus | ephippium |
| Brachycephalus | ferruginus |
| Brachycephalus | garbeanus |
| Brachycephalus | guarani |
| Brachycephalus | hermogenesi |
| Brachycephalus | izecksohni |
| Brachycephalus | margaritatus |
| Brachycephalus | nodoterga |
| Brachycephalus | pernix |
| Brachycephalus | pitanga |
| Brachycephalus | pombali |
| Brachycephalus | pulex |
| Brachycephalus | toby |
| Brachycephalus | tridactylus |
| Brachycephalus | vertebralis |
| Ceratophrys | aurita |
| Chiasmocleis | alagoanus |
| Chiasmocleis | atlantica |
| Chiasmocleis | capixaba |
| Chiasmocleis | carvalhoi |
| Chiasmocleis | cordeiroi |
| Chiasmocleis | crucis |
| Chiasmocleis | gnoma |
| Chiasmocleis | leucosticta |
| Chiasmocleis | mantiqueira |
| Chiasmocleis | sapiranga |
| Chiasmocleis | schubarti |
| Crossodactylodes | bokermanni |
| Crossodactylodes | izecksohni |
| Crossodactylodes | pintoi |
| Crossodactylus | aeneus |
| Crossodactylus | bokermanni |
| Crossodactylus | caramaschii |
| Crossodactylus | cyclospinus |
| Crossodactylus | dantei |
| Crossodactylus | dispar |
| Crossodactylus | gaudichaudii |
| Crossodactylus | grandis |
| Crossodactylus | lutzorum |
| Crossodactylus | schmidti |
| Crossodactylus | trachystomus |
| Cycloramphus | acangatan |
| Cycloramphus | asper |
| Cycloramphus | bandeirensis |
| Cycloramphus | bolitoglossus |
| Cycloramphus | boraceiensis |
| Cycloramphus | brasiliensis |
| Cycloramphus | carvalhoi |
| Cycloramphus | catarinensis |
| Cycloramphus | cedrensis |
| Cycloramphus | diringshofeni |
| Cycloramphus | dubius |
| Cycloramphus | duseni |
| Cycloramphus | eleutherodactylus |
| Cycloramphus | faustoi |
| Cycloramphus | fuliginosus |
| Cycloramphus | granulosus |
| Cycloramphus | izecksohni |
| Cycloramphus | juimirim |
| Cycloramphus | lithomimeticus |
| Cycloramphus | lutzorum |
| Cycloramphus | migueli |
| Cycloramphus | mirandaribeiroi |
| Cycloramphus | ohausi |
| Cycloramphus | organensis |
| Cycloramphus | rhyakonastes |
| Cycloramphus | semipalmatus |
| Cycloramphus | stejnegeri |
| Cycloramphus | valae |
| Dasypops | schirchi |
| Dendrophryniscus | berthalutzae |
| Dendrophryniscus | brevipollicatus |
| Dendrophryniscus | carvalhoi |
| Dendrophryniscus | krausae |
| Dendrophryniscus | leucomystax |
| Dendrophryniscus | oreites |
| Dendrophryniscus | organensis |
| Dendrophryniscus | skuki |
| Dendrophryniscus | stawiarskyi |
| Dendropsophus | anceps |
| Dendropsophus | berthalutzae |
| Dendropsophus | bipunctatus |
| Dendropsophus | branneri |
| Dendropsophus | decipiens |
| Dendropsophus | dutrai |
| Dendropsophus | elegans |
| Dendropsophus | giesleri |
| Dendropsophus | haddadi |
| Dendropsophus | limai |
| Dendropsophus | meridianus |
| Dendropsophus | microps |
| Dendropsophus | minutus |
| Dendropsophus | nahdereri |
| Dendropsophus | nanus |
| Dendropsophus | novaisi |
| Dendropsophus | oliveirai |
| Dendropsophus | pseudomeridianus |
| Dendropsophus | ruschii |
| Dendropsophus | sanborni |
| Dendropsophus | seniculus |
| Dendropsophus | soaresi |
| Dendropsophus | studerae |
| Dendropsophus | werneri |
| Elachistocleis | cesarii |
| Elachistocleis | erythrogaster |
| Eleutherodactylus | bilineatus |
| Euparkerella | brasiliensis |
| Euparkerella | cochranae |
| Euparkerella | robusta |
| Euparkerella | tridactyla |
| Fritziana | fissilis |
| Fritziana | goeldii |
| Fritziana | ohausi |
| Frostius | erythrophthalmus |
| Frostius | pernambucensis |
| Gastrotheca | albolineata |
| Gastrotheca | ernestoi |
| Gastrotheca | fissipes |
| Gastrotheca | flamma |
| Gastrotheca | fulvorufa |
| Gastrotheca | megacephala |
| Gastrotheca | microdiscus |
| Gastrotheca | prasina |
| Gastrotheca | pulchra |
| Gastrotheca | recava |
| Haddadus | binotatus |
| Holoaden | bradei |
| Holoaden | luederwaldti |
| Holoaden | pholeter |
| Hylodes | amnicola |
| Hylodes | asper |
| Hylodes | babax |
| Hylodes | cardosoi |
| Hylodes | charadranaetes |
| Hylodes | dactylocinus |
| Hylodes | glaber |
| Hylodes | heyeri |
| Hylodes | lateristrigatus |
| Hylodes | magalhaesi |
| Hylodes | meridionalis |
| Hylodes | mertensi |
| Hylodes | nasus |
| Hylodes | ornatus |
| Hylodes | otavioi |
| Hylodes | perere |
| Hylodes | perplicatus |
| Hylodes | phyllodes |
| Hylodes | pipilans |
| Hylodes | regius |
| Hylodes | sazimai |
| Hylodes | vanzolinii |
| Hyophryne | histrio |
| Hypsiboas | albomarginatus |
| Hypsiboas | albopunctatus |
| Hypsiboas | atlanticus |
| Hypsiboas | bischoffi |
| Hypsiboas | caingua |
| Hypsiboas | caipora |
| Hypsiboas | crepitans |
| Hypsiboas | curupi |
| Hypsiboas | cymbalum |
| Hypsiboas | exastis |
| Hypsiboas | faber |
| Hypsiboas | freicanecae |
| Hypsiboas | guentheri |
| Hypsiboas | joaquini |
| Hypsiboas | latistriatus |
| Hypsiboas | leptolineatus |
| Hypsiboas | marginatus |
| Hypsiboas | pardalis |
| Hypsiboas | poaju |
| Hypsiboas | polytaenius |
| Hypsiboas | pombali |
| Hypsiboas | prasinus |
| Hypsiboas | pulchellus |
| Hypsiboas | punctatus |
| Hypsiboas | raniceps |
| Hypsiboas | secedens |
| Hypsiboas | semiguttatus |
| Hypsiboas | semilineatus |
| Hypsiboas | stellae |
| Hypsiboas | stenocephalus |
| Ischnocnema | abdita |
| Ischnocnema | bolbodactyla |
| Ischnocnema | concolor |
| Ischnocnema | epipeda |
| Ischnocnema | erythromera |
| Ischnocnema | gehrti |
| Ischnocnema | gualteri |
| Ischnocnema | guentheri |
| Ischnocnema | henselii |
| Ischnocnema | hoehnei |
| Ischnocnema | holti |
| Ischnocnema | izecksohni |
| Ischnocnema | juipoca |
| Ischnocnema | karst |
| Ischnocnema | lactea |
| Ischnocnema | manezinho |
| Ischnocnema | melanopygia |
| Ischnocnema | nasuta |
| Ischnocnema | nigriventris |
| Ischnocnema | octavioi |
| Ischnocnema | oea |
| Ischnocnema | paranaensis |
| Ischnocnema | parva |
| Ischnocnema | pusilla |
| Ischnocnema | randorum |
| Ischnocnema | sambaqui |
| Ischnocnema | spanios |
| Ischnocnema | venancioi |
| Ischnocnema | verrucosa |
| Ischnocnema | vizottoi |
| Itapotihyla | langsdorffii |
| Leptodactylus | cupreus |
| Leptodactylus | flavopictus |
| Leptodactylus | furnarius |
| Leptodactylus | fuscus |
| Leptodactylus | gracilis |
| Leptodactylus | hylodes |
| Leptodactylus | jolyi |
| Leptodactylus | labyrinthicus |
| Leptodactylus | latrans |
| Leptodactylus | mystaceus |
| Leptodactylus | mystacinus |
| Leptodactylus | natalensis |
| Leptodactylus | notoaktites |
| Leptodactylus | plaumanni |
| Leptodactylus | podicipinus |
| Leptodactylus | spixi |
| Leptodactylus | troglodytes |
| Leptodactylus | vastus |
| Leptodactylus | viridis |
| Limnomedusa | macroglossa |
| Lithobates | palmipes |
| Macrogenioglottus | alipioi |
| Megaelosia | apuana |
| Megaelosia | bocainensis |
| Megaelosia | boticariana |
| Megaelosia | goeldii |
| Megaelosia | jordanensis |
| Megaelosia | lutzae |
| Megaelosia | massarti |
| Melanophrynicsus | vilavelhensis |
| Melanophryniscus | admirabilis |
| Melanophryniscus | alipioi |
| Melanophryniscus | cambaraensis |
| Melanophryniscus | dorsalis |
| Melanophryniscus | macrogranulosus |
| Melanophryniscus | moreirae |
| Melanophryniscus | peritus |
| Melanophryniscus | setiba |
| Melanophryniscus | simplex |
| Melanophryniscus | spectabilis |
| Melanophryniscus | tumifrons |
| Myersiella | microps |
| Odontophrynus | americanus |
| Odontophrynus | carvalhoi |
| Odontophrynus | maisuma |
| Paratelmatobius | cardosoi |
| Paratelmatobius | gaigeae |
| Paratelmatobius | lutzii |
| Paratelmatobius | mantiqueira |
| Paratelmatobius | poecilogaster |
| Paratelmatobius | yepiranga |
| Phasmahyla | cochranae |
| Phasmahyla | cruzi |
| Phasmahyla | exilis |
| Phasmahyla | guttata |
| Phasmahyla | jandaia |
| Phasmahyla | spectabilis |
| Phasmahyla | timbo |
| Phrynomedusa | appendiculata |
| Phrynomedusa | bokermanni |
| Phrynomedusa | fimbriata |
| Phrynomedusa | marginata |
| Phrynomedusa | vanzolinii |
| Phyllodytes | acuminatus |
| Phyllodytes | edelmoi |
| Phyllodytes | gyrinaethes |
| Phyllodytes | kautskyi |
| Phyllodytes | luteolus |
| Phyllodytes | maculosus |
| Phyllodytes | melanomystax |
| Phyllodytes | punctatus |
| Phyllodytes | tuberculosus |
| Phyllodytes | wuchereri |
| Phyllomedusa | bahiana |
| Phyllomedusa | burmeisteri |
| Phyllomedusa | distincta |
| Phyllomedusa | iheringii |
| Phyllomedusa | nordestina |
| Phyllomedusa | rohdei |
| Phyllomedusa | tetraploidea |
| Physalaemus | aguirrei |
| Physalaemus | atlanticus |
| Physalaemus | barrioi |
| Physalaemus | bokermanni |
| Physalaemus | caete |
| Physalaemus | camacan |
| Physalaemus | crombiei |
| Physalaemus | cuvieri |
| Physalaemus | erikae |
| Physalaemus | erythros |
| Physalaemus | feioi |
| Physalaemus | insperatus |
| Physalaemus | irroratus |
| Physalaemus | jordanensis |
| Physalaemus | kroyeri |
| Physalaemus | lateristriga |
| Physalaemus | lisei |
| Physalaemus | maculiventris |
| Physalaemus | maximus |
| Physalaemus | moreirae |
| Physalaemus | nanus |
| Physalaemus | obtectus |
| Physalaemus | olfersii |
| Physalaemus | orophilus |
| Physalaemus | signifer |
| Physalaemus | soaresi |
| Physalaemus | spiniger |
| Pipa | carvalhoi |
| Pristimantis | paulodutrai |
| Pristimantis | ramagii |
| Pristimantis | vinhai |
| Proceratophrys | appendiculata |
| Proceratophrys | avelinoi |
| Proceratophrys | bigibbosa |
| Proceratophrys | boiei |
| Proceratophrys | brauni |
| Proceratophrys | cristiceps |
| Proceratophrys | laticeps |
| Proceratophrys | melanopogon |
| Proceratophrys | moehringi |
| Proceratophrys | paviotii |
| Proceratophrys | phyllostoma |
| Proceratophrys | renalis |
| Proceratophrys | sanctaritae |
| Proceratophrys | schirchi |
| Proceratophrys | subguttata |
| Pseudis | bolbodactyla |
| Pseudis | cardosoi |
| Pseudis | fusca |
| Pseudis | minuta |
| Pseudopaludicola | falcipes |
| Rhinella | abei |
| Rhinella | achavali |
| Rhinella | crucifer |
| Rhinella | dorbignyi |
| Rhinella | fernandezae |
| Rhinella | granulosa |
| Rhinella | henseli |
| Rhinella | hoogmoedi |
| Rhinella | icterica |
| Rhinella | jimi |
| Rhinella | ornata |
| Rhinella | pygmaea |
| Rhinella | schneideri |
| Scinax | agilis |
| Scinax | albicans |
| Scinax | alter |
| Scinax | angrensis |
| Scinax | arduous |
| Scinax | argyreornatus |
| Scinax | ariadne |
| Scinax | aromothyella |
| Scinax | atratus |
| Scinax | auratus |
| Scinax | belloni |
| Scinax | brieni |
| Scinax | caldarum |
| Scinax | cardosoi |
| Scinax | carnevallii |
| Scinax | catharinae |
| Scinax | crospedospilus |
| Scinax | cuspidatus |
| Scinax | duartei |
| Scinax | eurydice |
| Scinax | flavoguttatus |
| Scinax | fuscomarginatus |
| Scinax | fuscovarius |
| Scinax | granulatus |
| Scinax | hayii |
| Scinax | heyeri |
| Scinax | hiemalis |
| Scinax | humilis |
| Scinax | imbegue |
| Scinax | insperatus |
| Scinax | juncae |
| Scinax | jureia |
| Scinax | kautskyi |
| Scinax | littoralis |
| Scinax | littoreus |
| Scinax | longilineus |
| Scinax | luizotavioi |
| Scinax | machadoi |
| Scinax | melloi |
| Scinax | obtriangulatus |
| Scinax | pachycrus |
| Scinax | peixotoi |
| Scinax | perereca |
| Scinax | perpusillus |
| Scinax | ranki |
| Scinax | rizibilis |
| Scinax | similis |
| Scinax | squalirostris |
| Scinax | strigilatus |
| Scinax | trapicheiroi |
| Scinax | tupinamba |
| Scinax | tymbamirim |
| Scinax | uruguayus |
| Scinax | v-signatus |
| Scinax | x-signatus |
| Scythrophrys | sawayae |
| Sphaenorhynchus | botocudo |
| Sphaenorhynchus | bromelicola |
| Sphaenorhynchus | caramaschii |
| Sphaenorhynchus | mirim |
| Sphaenorhynchus | orophilus |
| Sphaenorhynchus | palustris |
| Sphaenorhynchus | pauloalvini |
| Sphaenorhynchus | planicola |
| Sphaenorhynchus | prasinus |
| Sphaenorhynchus | surdus |
| Stereocyclops | incrassatus |
| Stereocyclops | palmipes |
| Stereocyclops | parkeri |
| Thoropa | lutzi |
| Thoropa | miliaris |
| Thoropa | petropolitana |
| Thoropa | saxatilis |
| Trachycephalus | atlas |
| Trachycephalus | dibernardoi |
| Trachycephalus | imitatrix |
| Trachycephalus | lepidus |
| Trachycephalus | mesophaeus |
| Trachycephalus | nigromaculatus |
| Trachycephalus | typhonius |
| Vitreorana | eurygnathum |
| Vitreorana | parvulum |
| Vitreorana | uranoscopum |
| Xenohyla | eugenioi |
| Xenohyla | truncata |
| Zachaenus | carvalhoi |
| Zachaenus | parvulus |
